# Effect of a Pragmatic eHealth Behavioral Gestational Weight Gain Intervention on Household Chaos in Pregnant People of Lower Socioeconomic Status: Randomized Controlled Trial

**DOI:** 10.2196/74146

**Published:** 2026-01-08

**Authors:** Chelsea L Kracht, Kaja Falkenhain, Emily W Flanagan, Abby D Altazan, Hannah E Cabre, Maryam Kebbe, Emily K Woolf, Robbie Beyl, Daniel S Hsia, John W Apolzan, Leanne Redman

**Affiliations:** 1 Department of Internal Medicine University of Kansas Medical Center Kansas City, KS United States; 2 Clinical Sciences Division Pennington Biomedical Research Center Baton Rouge, LA United States; 3 University of New Brunswick Fredericton Canada

**Keywords:** pregnancy, stress, disadvantaged, depression, anxiety

## Abstract

**Background:**

Household chaos is an emerging risk factor for childhood obesity development, especially in families with lower socioeconomic status (SES). It is unclear if changes in household chaos, especially in pregnancy, may mediate the effectiveness of weight-related behavioral interventions.

**Objective:**

This study aimed to describe how household chaos changed across gestation and determine whether household chaos mediated the effect of an eHealth behavioral gestational weight gain (GWG) intervention in pregnant people with low SES.

**Methods:**

Pregnant people who were enrolled in the US Special Supplemental Nutrition Program for Women, Infants, and Children (WIC) were recruited for a randomized controlled trial testing the effectiveness of an eHealth-based pragmatic intervention for GWG management. The usual care group received the standard WIC program guidance and monthly health coach support with general pregnancy recommendations. The intervention group received the standard WIC program plus health information via email and weekly health coach discussions to promote healthy eating and adequate physical activity. Weight and household chaos were measured at baseline (early pregnancy, 10^+0^ to 16^+6^ weeks gestation) and at the end of the intervention (late pregnancy, 35^+0^ to 37^+6^ weeks gestation). Household chaos changes across time were examined using a paired *t* test for the continuous score and using the McNemar test for household chaos category (improved or no change vs declined). Serial linear regression models and mediation analyses assessed the relationship between the intervention group (predictor), household chaos change (mediator), and GWG (outcome) with adjustment for covariates.

**Results:**

Among 258 participants, 53.9% (n=139) were Black, 43.4% (n=112) were nulliparous, 36.0% (n=93) were obese, and almost half (n=124, 48.1%) were classified as low household chaos at baseline. Overall, there were minimal changes in household chaos scores from early to late pregnancy (*P*=.34), although scores and categories tended to be higher in late pregnancy. Household chaos changes were divided; some improved or had no change (n=140, 54.3%), and some declined (n=118, 45.7%) across gestation. Household chaos did not mediate the effect of the intervention on GWG.

**Conclusions:**

In this sample, household chaos did not change across gestation and did not explain the effect of an eHealth behavioral GWG intervention in pregnant people with lower SES. Routine-focused and multilevel interventions may improve upon these findings to support an organized home for future parent and child health.

**Trial Registration:**

ClinicalTrials.gov NCT04028843; https://www.clinicaltrials.gov/study/NCT04028843

## Introduction

The early pregnancy environment can shape the development and future health of a child [1]. Maternal stress, either acute or chronic, may negatively impact the intrauterine environment through increasing allostatic load, which leads to physical and neurological consequences [2]. Maternal stress, coupled with poor mental health, has established negative implications for adverse pregnancy outcomes [3], poor child cognition [4], and overweight and obesity in adult offspring [5]. Individuals from lower socioeconomic statuses (SES) may have a heightened risk for prenatal stress due to continuous exposure to known stressors [6] such as economic and housing instability [7]. Innovative approaches to lessen maternal stress and improve mental health among populations facing economic disadvantages may help improve long-term maternal and child health.

Interventions during pregnancy to improve maternal mental health have mixed or null results, with some promising interventions using mindfulness approaches [8]. However, when considering stress, two separate systematic reviews of 41-44 studies documented that eHealth interventions were effective at improving pregnant people’s stress [9], including people with lower SES [10]. Lifestyle interventions for gestational weight gain (GWG) management have the potential to aid in establishing routine and healthy lifestyle behaviors and, in turn, reduce stress. A GWG intervention that trained health professionals improved anxiety in 205 pregnant people with obesity [11]. These results are likely attributable to the intervention’s focus on both diet and exercise, as a different randomized controlled trial providing supervised exercise training only did not improve maternal mental health [12]. Adopting a multibehavior lifestyle intervention for appropriate GWG to eHealth modalities may improve upon existing effective GWG interventions and reduce maternal stress, including in people with lower SES who are traditionally hard to reach, underserved, and have limited access to resources.

Household chaos is an established factor for poor child development and childhood obesity [13]. Household chaos differs from individual stress, as it evaluates stress at the home level; it is characterized by crowding, disorder, and noise in the home [14]. Higher levels of household chaos may negatively impact pregnant people’s ability to sustain lifestyle behaviors and manage stress, such as practicing mindfulness, having adequate physical activity, or creating a routine within the day [15]. Making individual changes such as reducing screen time and prioritizing healthy lifestyle behaviors (eg, sleep and family meals) may improve household chaos as demonstrated in a randomized pilot study [16]. These healthy lifestyle behavior improvements align with common elements of GWG interventions. However, a systematic scoping review of 111 studies found no studies examining household chaos that were conducted in pregnant people [13]. Household chaos literature has primarily focused on parents with young children [13,15,16], and existing evaluations in pregnancy have used household chaos as a covariate [17], identifying a significant knowledge gap. There is potential for household chaos to increase across gestation as the mother prepares for birth, and this increase may be offset by prenatal interventions targeting individual health behaviors to improve maternal and child health. Accordingly, an eHealth multicomponent lifestyle intervention to promote recommended GWG was delivered in pregnant people enrolled in the Special Supplemental Nutrition Program for Women, Infants, and Children (WIC) [18,19]. There was no significant difference in the incidence of appropriate GWG according to prepregnancy BMI-specific National Academy of Medicine 2009 guidelines. However, the intervention group demonstrated lower study-observed and weekly GWG as well as lower deviation from GWG guidelines relative to the control group. This paper describes the results of a preplanned, but not preregistered, secondary analysis using data collected from the trial [19], thereby filling a gap in the literature by examining household chaos across gestation in the context of an evidence-based lifestyle intervention. Accordingly, this study seeks to (1) describe changes in household chaos and stress-related constructs across pregnancy in a lower SES population and (2) examine if changes in household chaos mediate the effect of an eHealth intervention on appropriate GWG in a lower SES population. We hypothesized that household chaos would increase across gestation (aim 1); further, household chaos changes would negatively impact and mediate the effect of a lifestyle intervention on GWG (aim 2).

## Methods

### Participants and Procedures

Pregnant people enrolled in WIC within the US state of Louisiana were recruited during 2019-2023 for a 2-arm parallel design randomized controlled trial aimed at increasing adherence to GWG recommendations [20]. In brief, women were eligible to enroll if they had a singleton viable pregnancy, were WIC recipients for their current pregnancy, were less than 16 weeks gestational age, had a BMI between 18.5 and 40.0 kg/m^2^, had access to a smartphone with internet access, and were willing to be identified on social media to other participants. Exclusion criteria were ages younger than 18 years or older than 40 years, current drug use (including tobacco and alcohol), non–pregnancy-related chronic disease (cancer, heart disease, HIV, or type 1 or type 2 diabetes), hypertension (systolic blood pressure >160 mm Hg or diastolic blood pressure >110 mm Hg at screening), current unstable mental health or an eating disorder, plan to move out of the state less than 1 year post partum, or inability or unwillingness to complete a run-in period task of keeping an activity diary with at least 80% compliance. Eligible participants were randomized (stratified by their respective state region and BMI category) to either a behavioral intervention for appropriate GWG (alongside usual WIC services; intervention group) or to receive usual WIC services only (usual care group). The trial was designed to test the hypothesis that individuals in the intervention group would have a higher incidence of appropriate GWG (as defined by the National Academy of Medicine) relative to the usual care group [21]. Details of the study protocol are further described elsewhere [19].

This secondary analysis used data collected at the baseline visit in early pregnancy (10^+0^ to 16^+6^ weeks gestation) and at the end-of-intervention visit in late pregnancy (35^+0^ to 37^+6^ weeks gestation). At baseline, participants completed a demographic questionnaire and other questionnaires related to mental health, and paper forms were entered into a secure online platform [22]. At the end-of-intervention visit, participants repeated the mental health questionnaires in the same manner. Anthropometrics were assessed in-person at both visits.

### Intervention Groups

In brief, the behavioral intervention (“Healthy Beginnings”) was an approximately 24-week eHealth intervention focused on self-monitoring of weight and weight-related health behaviors and included personalized feedback from a trained health coach [19]. The intervention consisted of weekly lessons in the form of short videos and content related to adequate physical activity, healthy eating habits, and self-monitoring of weight based on evidence-based practices. The lessons were supplemented with weekly individual coaching check-ins, a closed Facebook group to interact with other study participants, and rewards for engaging in the intervention (eg, watching videos and self-monitoring weight). Rewards could be redeemed for pregnancy and postpartum-related items (eg, diapers). Participants also received a cellular-enabled scale and a Fitbit to promote self-monitoring and data from which coaches used as additional tools for individual counseling sessions. Topics specific to routine and stress included lessons on time management, meal preparation, behavior chains, and building social support, which all occurred in the first 8 lessons, while later lessons (week 17-24) included information on mindfulness and relaxation techniques, stress and sleep, and postpartum depression (Table S1 in Multimedia Appendix 1). No major changes to eligibility, version content, bugs, or content occurred during the study.

The usual care group received standard WIC services, which included general weight management advice, and received monthly check-ins with a health coach to encourage study retention. Closed Facebook groups included pregnancy-related topics other than physical exercise and healthy eating (ie, non–weight-related).

### Household Chaos

The Confusion, Hubbub, and Order Scale was used to assess household chaos in early and late pregnancy and was previously validated in mothers of young children [14]. This 15-item questionnaire investigates agreement with statements related to the participant’s current household, including disorder, crowding, and noise. Questions include four Likert scale response options, ranging from “strongly disagree” to “strongly agree,” with reverse coding for 8 questions. Responses were summed, and total scores ranged from 15 to 60, with a higher score indicating a more chaotic home. The total score was further categorized based on previous investigations of household chaos into 4 categories: low (score <25), moderate to low (score 25-30), moderate to high (score 31-35), and high (score >35) [23]. Change in household chaos across gestation was calculated by subtracting early pregnancy scores from late pregnancy scores.

### Gestational Weight Gain

Height and weight were measured during study visits by study staff before randomization in early pregnancy, and weight was measured again in late pregnancy. Height was measured using a portable stadiometer, and weight was measured twice using a standardized, calibrated electronic scale (Tanita Corp) to the nearest 0.1 kg with the participant wearing light clothing. BMI was calculated using the standard formula (kg/m^2^). GWG was calculated by subtracting early pregnancy weight from late pregnancy weight.

### Covariates

We explored potential covariates for inclusion in statistical models based on past literature on maternal stress and household chaos, which included demographics, other mental health constructs, and gestational diabetes status [11,12]. At baseline, participants completed a questionnaire on their race or ethnicity, marital status, and parity. Participants also completed the 21-item Depression Anxiety Stress Scales (DASS-21), a questionnaire containing statements related to subscales of depression, anxiety, and stress [24]. Participants rated how much a statement applied to them over the prior week with 4 response options ranging from “never” to “always.” Subscale questions were summed, with a higher score indicating more frequent symptoms in that subscale. Consistent with another study of prenatal stress and household chaos [17], baseline stress was included as a covariate in our statistical models to isolate the effect of household chaos. Gestational diabetes status was obtained through abstraction from birth certificate records received from the state following delivery in late pregnancy. Sleep duration was measured using an accelerometer (ActiGraph GT3x+) placed on the nondominant wrist and worn for 7 days during early and late pregnancy. Overnight sleep time was identified using GGIR version 3.0.5 [25]; this R package uses an algorithm that identifies sleep based on raw accelerometer data [26,27].

### Statistical Analysis

Participants in both the intervention group and the usual care group who had data for early and late pregnancy household chaos, GWG, and covariates were retained for analysis. Frequencies and means of baseline characteristics were calculated for the entire sample and compared by intervention group using chi-square or independent-sample *t* tests for categorical and continuous data, respectively.

For the first aim, a paired *t* test was used to determine changes in household chaos across gestation using the entire sample. We also explored changes in depression, anxiety, and stress as measured via the DASS-21 [24]. Household chaos category changes across time were compared using a McNemar test. Household chaos change score was categorized as improvement or no change (score change ≤0) or worsening (score change >0). The no change group was combined with the improvement group, as this may be considered another positive indicator of household chaos, since no changes were seen across a stressful time (ie, pregnancy). Identified covariates were compared between these change groups. To further describe the sample and changes across pregnancy, we also examined within-group changes among BMI categories (normal weight, overweight, obesity) and treatment groups using paired *t* tests. Within-group comparisons of early pregnancy, late pregnancy, and change in household chaos variables with weight-related variables were examined using Pearson correlations.

For the second aim, the mediation model of intervention (predictor), household chaos change (mediator), and GWG (outcome) were tested. Given that the primary paper demonstrates an intervention effect on GWG [18], this analysis was not presented. Covariates that were associated with baseline household chaos (*P*<.20) were retained, including race and ethnicity (non-Hispanic White, non-Hispanic Black, Hispanic, and mixed or other), parity (categories 0, 1, 2+), BMI category at randomization, time of enrollment with regard to COVID-19 (before March 2020, March 2020-March 2021, April 2021, and after), and gestational diabetes status (yes or no; Table S2 in Multimedia Appendix 1). Time of enrollment registered slightly above our threshold, but was retained due to existing literature of higher chaos in low-income families during this period [28]. Baseline household chaos was significantly correlated with all 3 subscales of the DASS-21 (Table S3 in Multimedia Appendix 1), and the stress scale was chosen as a covariate to align with past investigations of household chaos and stress [15]. For mediation, we conducted a linear regression model of calculated household chaos change with the intervention’s fixed effect and adjustment for early pregnancy stress, race and ethnicity, parity, BMI category, time of enrollment, gestational diabetes status, and early pregnancy household chaos. Then we conducted two more linear regression models: (1) the calculated GWG with the fixed effect of the intervention group, and (2) the calculated household chaos changes with the outcome of the calculated GWG using a similar approach and adjustment for the same covariates. Those models retained the same predictor, outcome, and covariates for adjustment. This approach was conducted with the PROCESS vs3.5 macro with 10,000 bootstrap intervals with unstandardized estimates [29]. Analyses were conducted using R statistical software (R Foundation for Statistical Computing) and SAS (version 9.4; SAS Institute), and statistical significance was set at *P* less than .05.

### Ethical Considerations

Pennington Biomedical Research Center provided institutional review board and ethics approval (2018-039-PBRC). Written informed consent was obtained at the early pregnancy visit, and from all participants in the study prior to study procedures. The informed consent specified that every effort would be made to maintain participant confidentiality, including through deidentifying private information. Participants were compensated US $25 for each study visit and up to US $150 for completion of all study visits.

## Results

### Overview

**Figure 1:**
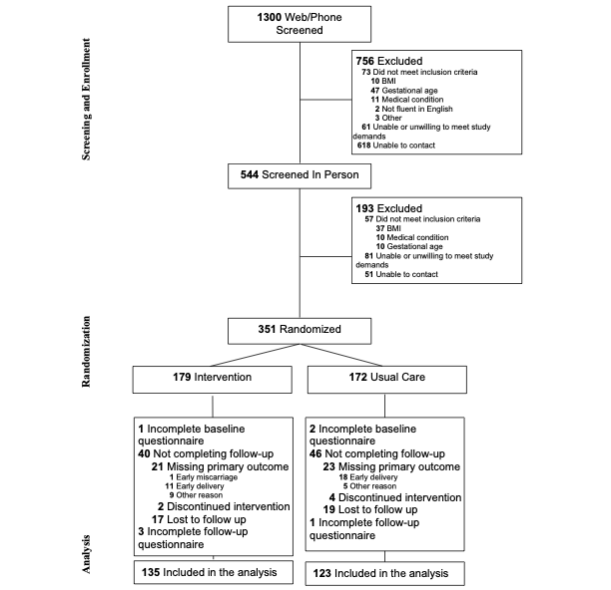
CONSORT diagram for illustrating participant progression through the trial.

The trial randomized 351 pregnant people, and 348 had complete baseline data (Tables S4 and S5 in Multimedia Appendix 1). The 348 individuals were reduced to 258 individuals for analysis, as 85 individuals did not complete the final visit and 5 did not have late pregnancy household chaos scores (Figure 1). There was no difference in early pregnancy household chaos score between those included (mean 25.9, SD 7.4; n=258) and those not included due to missing data (mean 26.6, SD 9.2; n=60).

As shown in Table 1, there was a comparable distribution across treatment groups. Participants were primarily non-Hispanic Black (139/258, 53.9%), married or living with a significant other (147/258, 57.0%), and nulliparous (112/258, 43.4%). There was a significant difference in the parity distribution between treatment groups (*P*=.02), but no other differences in maternal characteristics or health outcomes (All *P*>.05).

**Table 1:** Demographic characteristics of included pregnant people (N=258).

Maternal characteristics			All (N=258)		Intervention (n=135)			Usual care (n=123)		*P* value^a^	
Maternal age (years), mean (SD)			27.5 (6.0)		27.5 (6.0)			27.5 (6.0)		.99	
Weight (kg), mean (SD)			74.8 (16.9)		74.2 (16.4)			75.3 (16.5)		.58	
**BMI (kg/m^2^), mean (SD)**			32.2 (5.0)		31.8 (5.0)			32.6 (5.0)		.18	
	Normal (18.5-24.9), n (%)	89 (34.5)		47 (34.8)			42 (34.1)		.99		
	Overweight (25-29.9), n (%)	76 (29.5)		36 (26.7)			40 (29.6)		—^b^		
	Obese (30-40), n (%)	93 (36.0)		48 (35.6)			45 (36.6)		—		
**Race and ethnicity, n (%)**											.35
	Hispanic	18 (7.0)		7 (5.2)			11 (8.9)				
	Non-Hispanic White	85 (32.9)		45 (33.3)			40 (32.5)				
	Non-Hispanic Black	139 (53.9)		77 (57.0)			62 (50.4)				
	Mixed or other	16 (6.2)		6 (4.4)			10 (8.1)				
**Marital status, n (%)**											.30
	Married/living with significant other	147 (57.0)		81 (60.0)			66 (53.7)				
	Not married	111 (43.0)		54 (40.0)			57 (46.3)				
**Education level, n (%)**											.91
	Postgraduate work	9 (3.5)		4 (3.0)			5 (4.1)				
	College degree	31 (12.0)		17 (12.6)			14 (11.4)				
	1-3 years of college or technical school	128 (49.6)		64 (47.4)			64 (52.0)				
	High school diploma or equivalent	74 (28.7)		41 (30.4)			33 (26.8)				
	Some high school	16 (6.2)		9 (6.7)			7 (5.7)				
**Parity, n (%)**											.02^c^
	0	112 (43.4)				63 (46.7)	49 (39.8)				
	1	69 (26.7)				27 (20.0)	42 (34.1)				
	2	34 (13.2)				24 (17.8)	10 (8.1)				
	3+	43 (16.7)				21 (15.6)	22 (17.9)				
**Enrollment time, mean (SD)**											.70
	Pre–COVID-19 (2019-March 2020)	15 (5.8)		8 (5.9)			7 (5.7)				
	COVID-19 (March 2020-March 2021)	41 (15.9)		19 (14.1)			22 (17.9)				
	Post–COVID-19 (April 2021-2023)	202 (78.3)		108 (80.0)			94 (76.4)				
Gestational diabetes, n (%)			19 (7.4)		9 (6.7)			10 (8.1)		.68	
Baseline stress, mean (SD)			9.7 (8.7)		9.7 (8.9)			9.3 (8.2)		.69	

^a^Comparisons between groups were conducted using chi-square (categorical variables) or linear regression (continuous variables).

^b^Not applicable.

^c^*P*<.05.

### Aim 1: Household Chaos Across Pregnancy

Across both treatment groups combined, most individuals were classified as having low (score <25, n=124, 48.1%) or moderate or low (score 25-30, n=73, 28.3%) household chaos in early pregnancy (Table 2). There were minimal changes in household chaos scores from early to late pregnancy, although scores and categories tended to be higher in late pregnancy (Table 2). Similarly, there were no significant changes in depression or stress scores, although there was a small improvement in anxiety among the full sample (Table S6 in Multimedia Appendix 1).

**Table 2:** Changes in household chaos across gestation (n=258).

				Early pregnancy		Late pregnancy		Change			*P* value^a^	
Total score, mean (SD)				25.9 (7.4)		26.4 (8.5)		0.49 (8.27)			.34	
**Category, n (%)**									—^b^			.21
	Low (<25)			124 (48.1)		128 (49.6)						
	Moderate-low (25-30)			73 (28.3)		70 (27.1)						
	Moderate-high (31-35)			36 (14.0)		22 (8.5)						
	High (36-60)			25 (9.7)		37 (14.3)						
**Household chaos improved or no change (n=140)**												
	Total score, mean (SD)			27.9 (7.6)		23.4 (6.2)		–4.4 (5.0)			.01^c^	
	**Category, n (%)**							—			.01^c^	
		Low (<25)	49 (35.0)		86 (61.4)							
		Moderate-low (25-30)	48 (34.3)		40 (28.6)							
		Moderate-high (31-35)	22 (15.7)		6 (4.3)							
		High (36-60)	21 (15.0)		8 (5.7)							
**Household chaos worsened (n=118)**												
	Total score, mean (SD)			23.6 (6.3)		30.0 (9.4)		6.3 (7.4)			.01^c^	
	**Category, n (%)**							—			.01^c^	
		Low (<25)	75 (63.6)		42 (35.6)							
		Moderate-low (25-30)	25 (21.2)		30 (25.4)							
		Moderate-high (31-35)	14 (11.9)		16 (13.6)							
		High (36-60)	4 (3.4)		29 (24.6)							

^a^Comparisons between groups were conducted using the McNemar test (categorical variables) or paired *t* test (continuous variables).

^b^Not applicable.

^c^*P*<.05.

Participants were categorized into improvement groups: either household chaos improving or no change, or worsening (Table 2). Approximately half of the 258 participants (n=140, 54.3%) had their household chaos improve (n=109, 42.2%) or had no change (n=31, 12.0%), and the others had their household chaos worsen (n=118, 45.7%). Both improvement groups had a mean change of around 5 points, either an increase or decrease, which resulted in shifts across the 5-point categories (*P*<.05). Using two SDs of this population mean (14.60 units) [30], we found only 6.2% (n=16) participants reported changes more than two SDs, with 3.1% (n=8) of the sample reporting a clinically significant decrease in household chaos scores and 3.1% (n=8) of the reporting a clinically significant increase in household chaos scores. Participants with improved or no change in household chaos scores were mostly categorized in the moderate-to-low chaos category in early pregnancy, but were categorized as low chaos by late pregnancy scores (n=158, 61.2%). They also began with a higher (or worse) early pregnancy household chaos score (mean 27.9, SD 7.6) compared to those in the worsening group (mean 23.6, SD 6.3). The worsening group was the converse, whereby the majority began in the low chaos category and were categorized as moderate-to-low chaos category in late pregnancy. Only early pregnancy chaos was associated with the improvement category. There were no other significant relationships between early pregnancy covariates and household chaos improvement category or change (all *P*>.05).

Within BMI categories independent of treatment assignment, there were no significant changes in household chaos scores across time (all *P*>.05; Table S6 in Multimedia Appendix 1). In individuals who were overweight at enrollment (n=76), early pregnancy weight was positively correlated with early pregnancy household chaos scores (*r*=0.32; *P*=.004) and negatively correlated with household chaos change (*r*=–0.22; *P*=.05). Therefore, people with a higher weight within the overweight category reported a higher household chaos score in early pregnancy but lowered their chaos over time. There were no other significant associations between early pregnancy scores, household chaos change, and weight outcomes in BMI categories.

When examining within-treatment groups and not BMI categories, there were no significant changes in household chaos across time (all *P*>.05; Table S6 in Multimedia Appendix 1). In the intervention group (n=135), GWG was negatively associated with late pregnancy household chaos (*r*=–0.17; *P*=.03). Therefore, intervention group participants who gained less weight had a higher late pregnancy household chaos score. Similarly, in the intervention group, early pregnancy household chaos score was negatively associated with GWG (*r*=–0.13; *P*=.12), but this was not statistically significant. There were no other significant effects between household chaos scores at either time point or the change thereof and weight outcomes in treatment groups.

### Aim 2: Mediation Model of Intervention, Household Chaos, and Gestational Weight Gain

In the adjusted models, household chaos change did not differ by treatment (*P*=.74). Both early pregnancy household chaos (β=–0.53, SE 0.06; *P*=.001) and DASS-21–assessed stress (β=0.15, SE 0.05; *P*=.004) were associated with household chaos change in these models. Further, household chaos change was not associated with GWG (*P*=.96). Higher parity (β=–1.06, SE 0.40; *P*=.009), BMI category (β=–2.49, SE 0.39; *P*=.001), and gestational diabetes status (β=–2.66, SE 1.27; *P*=.03) was negatively associated with GWG (all *P*<.05). A mediation model was not pursued, as there was no association between the predictor and mediator or mediator and outcome.

## Discussion

### Principal Findings

The purpose of this paper was to examine household chaos across pregnancy in a sample of pregnant people with lower SES and to examine whether additional household chaos negatively impacted the effect of a behavioral lifestyle intervention on GWG. In this study, people began pregnancy with generally low or moderate-to-low household chaos and with differing, although minimal, progression across gestation. The individual-focused eHealth behavioral intervention did not change household chaos score, nor was the amount of household chaos or its change related to GWG.

To our knowledge, there are limited reports of household chaos during pregnancy [13]. The observed mean early and late pregnancy household chaos scores were comparable to lower-income mothers of 12-month-old infants (mean 25.1, SD 6.7) [31], but lower than parents of young children (score 31) [32]. These lower household chaos scores may be attributed to around half of the current sample (112/258, 43.4%) being nulliparous. Accordingly, a minority of the current sample was classified as “low” chaos at either point in gestation compared to other samples using similar chaos categories: parents of young children (27%) [23], and mothers of young children during the COVID-19 pandemic (20%) [33]. Moreover, fewer pregnant people in this sample were categorized as high chaos in early pregnancy (~11%) compared to these past investigations of primarily high-income parents of young children (range 24%-27%) [23,33]. This finding is surprising, given a mixed methods study in this same state found that the COVID-19 pandemic and hurricanes negatively impacted pregnant people’s home environment and their mental health, especially pregnant people with low SES [34]. Two immediate explanations may be presented. First, only pregnant people who live in homes with low chaos may enroll in such intensive lifestyle intervention due to additional support at home or time available; therefore, these findings may be a result of selection bias and lower early pregnancy chaos serving as a facilitator to participation. Further, people in high-chaos homes may have delivered early; indeed, distress during pregnancy is a known risk factor for preterm birth [35]. Even so, there was no difference in the early pregnancy household chaos score between those who were included and those not included in the analysis. The second consideration is that household chaos scores significantly increase after the birth of the child, which may align with the notable prevalence and impacts of postpartum depression [36]. This increase may be sustained as the child ages, as higher parity was associated with a higher early pregnancy household chaos score in this sample.

We did not find support for our first hypothesis that household chaos scores would increase across gestation; we did not observe changes in household chaos across gestation when explored continuously, and there was an even split of improvement and worsening of household chaos. The first consideration is that the finding could reflect regression to the mean, whereby the data may move closer to the mean after an extreme value. Still, these results echo minimal changes across 12 months (1 point) in a longitudinal investigation in infants of lower-income homes [31]. Using a two SD cutoff of the current sample, we found very few made clinically meaningful changes. However, these results assume that this sample is a functional or normal population [30]; yet, no such normative data exist in pregnancy. Therefore, it is unclear if the 5-point changes in both improvement and worsening groups could also be clinically meaningful, as other longitudinal investigations have primarily examined similar median mean splits [37] or used differing scoring for the current questionnaire [16,38,39]. Changes between improvement groups indicated that those with moderate-to-low chaos improved their environment, while individuals in the low household chaos category did not sustain their routine environment. After months of additional anxiety, parents may make changes to cope and reduce anxiety before the arrival of the baby. These changes mirror another longitudinal investigation whereby stress at 6 months post partum led to improvements in sleep duration at 8 months post partum [40]. Another consideration is that the explored covariates (eg, age at enrollment and parity) did not change as pregnancy progressed. Investigating time-varying covariates, such as income and employment changes, may improve upon these practices.

We did not find support for our second hypothesis, that household chaos change would mediate the relationship between the intervention and GWG. Rather, across household chaos categories and despite any household chaos change, pregnant people were able to gain less weight in the intervention. These null results align with another obesity-focused intervention in 77 families of young children (ages 18 months-5 years) that showed minimal change (~1 point) after a 6-month intervention and 1-year follow-up [32], but contrast a routine-focused intervention in 54 parents of young children (ages 2-4 years) that decreased household chaos within 12 weeks [16]. Others have hypothesized that the relationship between stress and GWG is primarily explained by demographic factors, like low SES and lower education [41,42]. Our analysis did find that early pregnancy stress was a key covariate between the intervention group and household chaos change. This may suggest that early pregnancy stress is a mediator of household chaos change. It is possible that early pregnancy stress may negatively impact household chaos or, in turn, lead to an increase in household chaos. This current sample’s common factor of low SES may build upon other higher-income GWG interventions, but may also preclude associations between stress and GWG. Examination of specific health behaviors across the intervention may further elucidate the mechanism of household chaos and gaining an appropriate amount of weight in pregnancy [43], as household chaos is directly linked to maternal sleep in mothers of young children [15]. In this intervention, it is possible that participants adopted a healthier lifestyle, as indicated by gaining less GWG, which may have positive implications for their future parenting practices and reduce the impact of household chaos on child development.

**Strengths and Limitations**

Strengths of this study include the innovative examination of household chaos across pregnancy, an evidence-based pragmatic eHealth lifestyles intervention that occurred within a vulnerable population, and the concurrent measurement of stress concepts. Limitations include the lack of assessment of other covariates, missing household chaos data for some individuals in late pregnancy, self-report of chaos measures, difficulty identifying active intervention components, and potential lack of generalizability to other pregnant populations. First, additional information on household and economic stability [7], including food insecurity [44], employment security, and family composition (ie, number of children), may help identify further contributors to household chaos in this population. Nevertheless, the current assessment of household chaos did capture parity, and the household chaos score was correlated with other measures of mental health in this study (eg, depression, anxiety, and stress), validating its assessment of poor mental health. Second, identifying active intervention ingredients was difficult as the intervention addressed critical concepts (eg, stress reduction, routine, and the home environment) in multiple intervention sessions. This multicomponent approach allowed for significant intervention effects on GWG [18], but it limited our ability to thoroughly examine intervention components on maternal mental health. Moreover, this analysis was conducted by group assignment, and further investigation into how higher or lower amounts of household chaos may impact engagement in the intervention is warranted. Third, these results are confined to a self-reported questionnaire, which provides the opportunity for social desirability bias to arise, potentially resulting in the reporting of lower household chaos scores. Given the intervention’s eHealth modality and the ensuing COVID-19 pandemic, an existing validated questionnaire was conducted using a paper form rather than delivered during an in-home or psychologist visit. Finally, the current investigation is confined to pregnant people without current unstable mental health conditions who were enrolled in a supplemental nutrition program in a southern US state. These results may not apply to pregnant people with current mental health disorders (eg, prenatal depression), not participating in WIC, or living in other US or global regions.

**Future Directions**

The current results suggest 4 major areas for future research, policy, and practice. First, household chaos assessment across pregnancy and postpartum may help identify critical time points for intervention and such normative data for clinically meaningful cutoffs. Second, evaluating the effect of in-person intervention on household chaos may improve upon the current study’s eHealth-based intervention. Though in-person interventions may result in lower compliance [12], they may foster higher social support and changes at home. Third, creating a household chaos program for pregnancy may still be warranted for high-chaos households or to prepare for the higher chaos when the baby arrives. This potential program may include focusing on household routines, reducing household screen time, and promoting family cohesion for parent and family stress reduction [16]. Moreover, reducing any household chaos in the postpartum period may help support maternal mental and physical health. Fourth, creating a multilevel program to improve economic and housing stability may reduce household stress and improve upon this individual-focused intervention. Even so, it may be seen as a benefit that the current intervention did not result in more household chaos in this population.

### Conclusion

In this sample of pregnant people with lower SES, a low level of household chaos was sustained across gestation. Accordingly, this intervention was related to lower GWG, but not their household chaos scores or change across 6 months. Beneficial changes in lifestyle behaviors have positive implications for adequate weight gain, reducing stress, and future positive parenting practices. Routine-focused and multilevel interventions may improve upon these findings to support an organized home for future parent and child health.

## Data Availability

The datasets used and/or analyzed during the current study are available from the corresponding author on reasonable request.

## Appendix

Multimedia Appendix 1 SmartMoms SmartTips by week, covariates of baseline household chaos, early pregnancy mental health correlations, participant demographics, and household chaos changes by BMI and treatment group.

Multimedia Appendix 2 CONSORT e-Health Checklist (V 1.6.1).

## Abbreviations

DASS-21: 21-item Depression, Anxiety, and Stress Scale
GWG: gestational weight gain
SES: socioeconomic status
WIC: Supplemental Nutrition Program for Women, Infants, and Children
