# Supplementary material for: Effect of a Pragmatic eHealth Behavioral Gestational Weight Gain Intervention on Household Chaos in Pregnant People of Lower Socioeconomic Status: Randomized Controlled Trial
Source: JMIR Ment Health. 2026 Jan 8;13:e74146. doi: 10.2196/74146 (PMC12828310; doi:10.2196/74146)
Supplement: Multimedia Appendix 1 [file mental_v13i1e74146_app1.docx]

**Table S1.** SmartMoms® SmartTips by week.

|  | |
| --- | --- |
| Week | General Topic |
| 1 | Weight graph and weight gain in pregnancy |
| 2 | Time management and overcoming barriers to success |
| 3 | Meal planning, grocery shopping and your WIC Food Package |
| 4 | Meal prep and healthy cooking |
| 5 | Portion control and eating patterns |
| 6 | Behavior chains |
| 7 | Controlling food cues and hunger |
| 8 | Building social support |
| 9 | Emotional eating |
| 10 | All you need to know about GDM |
| 11 | Protein and fat |
| 12 | Fluids and fiber |
| 13 | Carbohydrate and sugar |
| 14 | Prenatal Vitamins |
| 15 | Social eating (baby shower, church) |
| 16 | Importance of being active |
| 17 | Mindfulness and relaxation techniques |
| 18 | Managing food cravings and snacking |
| 19 | Healthy eating for moms on the go |
| 20 | Stress and sleep |
| 21 | Postpartum depression |
| 22 | Preparing for labor and birth* |
| 23 | Breastfeeding |
| 24 | Optimizing health postpartum |
|  | Congratulations & graduation! |

**Table S2.** Associations of potential covariates with baseline household chaos.

|  | Household Chaos |
| --- | --- |
|  | Estimate [95% CI] |
| Age, yrs | 0.0 [-0.2; 0.1], p=0.53 |
| Race/Ethnicity | p=0.06 |
| Non-Hispanic White  Non-Hispanic Black  Hispanic  Mixed or Other | -  -2.4 [-4.2; -0.5], P=0.01  -1.4 [-5.0; 2.2], P=0.45  -3.4 [-7.3; 0.5], P=0.09 |
| Parity, continuous | 0.8 [0.3; 1.3], P=0.003 |
| Parity, categorical | p=0.08 |
| 0  1  2+ | -  0.1 [-1.9; 2.2], P=0.90  2.1 [0.1; 4.1], P=0.04 |
| Marital status | p=0.62 |
| Married/Living with significant other  Not married | -  0.4 [-1.3; 2.1], P=0.62 |
| Body mass index, continuous | 0.1 [0.0; 0.3], P=0.14 |
| Body mass index, categorical | p=0.17 |
| Normal weight, 18.5-24.9, n (%)  Overweight, 25.0-29.9, n (%)  Obesity, 30.0-40.0, n (%) | -  -1.2 [-3.3; 0.9], P=0.27  0.8 [-1.2; 2.7], P=0.44 |
| Education | p=0.24 |
| College degree or post-graduate  1-3 yrs of college education  High school or some high school | -  -0.8 [-3.2; 1.6], P=0.51  0.8 [-1.6; 3.2], P=0.52 |
| Environment | p=0.26 |
| Rural  Urban | -  -1.1 [-3.1; 0.8], P=0.26 |
| Sleep (minutes/day) | 0.0 [0.0; 0.0], P=0.32 |
| Year of enrolment | p=0.33 |
| Before March 2020  March 2020 – March 2021  April 2021 and after | -  2.5 [-2.1; 7.0], p=0.29  2.9 [-1.0; 6.8], p=0.14 |
| Gestational diabetes | -2.4 [-5.6; 0.8], p=0.14 |
| *Data presented as coefficient with corresponding 95% confidence interval and P value derived from a linear model. For categorical predictors, the top category is the referent group. Sleep assessed using wrist-worn accelerometry and validated algorithms.* | |

**Table S3.** Correlations between early pregnancy mental health measures.

|  | Household Chaos | Depression | Anxiety | Stress |
| --- | --- | --- | --- | --- |
| Household Chaos | - |  |  |  |
| Depression | 0.34 [0.24; 0.43], p<0.001 | - |  |  |
| Anxiety | 0.27 [0.17; 0.37], p<0.001 | 0.68 [0.62; 0.73],  p<0.001 | - |  |
| Stress | 0.36 [0.27; 0.46].  p<0.001 | 0.77 [0.73; 0.81],  p<0.001 | 0.73 [0.68; 0.77],  p<0.001 | - |
| *Data presented as Spearman’s correlation coefficient with corresponding 95% confidence interval and p-value.* | | | | |

**Table S4.** Demographic characteristics of included pregnant people (n=348).

|  | | | | |
| --- | --- | --- | --- | --- |
|  | All  (n=351) | Intervention  (n=179) | Usual Care  (n=172) | P Value |
|  | Mean (SD) | Mean (SD) | Mean (SD) |  |
| *Maternal characteristics* |  |  |  |  |
| Maternal age, years | 27 (6) | 27 (6) | 28 (6) | 0.62 |
| Gestational age at randomization, weeks | 13 (2) | 13 (2) | 13 (2) | 0.93 |
| Weight, kg | 75.1 (16.9) | 74.8 (17.5) | 75.4 (16.4) | 0.75 |
| Body mass index, kg/m^2^ | 28.5 (5.8) | 28.6 (5.9) | 28.5 (5.7) | 0.92  0.99 |
| Normal, 18.5-24.9, n (%) | 114 (33) | 58 (32) | 56 (33) |  |
| Overweight, 25.0-29.9, n (%) | 99 (28) | 51 (29) | 48 (28) |  |
| Obesity, 30.0-40.0, n (%) | 138 (39) | 70 (39) | 68 (39) |  |
| Race/ethnicity, n (%) |  |  |  | 0.78 |
| Hispanic | 23 (7) | 10 (6) | 13 (8) |  |
| Non-Hispanic White | 109 (31) | 55 (31) | 54 (31) |  |
| Non-Hispanic Black | 201 (57) | 106 (59) | 95 (55) |  |
| Mixed or other | 18 (5) | 8 (4) | 10 (6) |  |
| Marital status, n (%) |  |  |  | 0.70 |
| Married/living with significant other | 200 (57) | 104 (58) | 96 (56) |  |
| Not married | 151 (43) | 75 (42) | 76 (44) |  |
| Education level, n (%) |  |  |  | 0.77 |
| College degree or post graduate work | 52 (15) | 29 (16) | 23 (13) |  |
| 1-3 yrs of college education or similar | 168 (48) | 80 (45) | 88 (51) |  |
| High school or some high school | 129 (37) | 69 (39) | 60 (35) |  |
| Parity, n (%) |  |  |  | 0.02* |
| 0 | 150 (43) | 84 (47) | 66 (38) |  |
| 1 | 93 (26) | 36 (20) | 57 (33) |  |
| 2 | 51 (15) | 32 (18) | 19 (11) |  |
| 3+ | 57 (16) | 27 (15) | 30 (18) |  |
| *Maternal Mental Health* |  |  |  |  |
| Household chaos, au, median (range) | 25 (15-54) | 24 (15-50) | 25 (15-54) | 0.31  0.56 |
| Low, <25, n (%) | 175 (50) | 95 (53) | 80 (47) |  |
| Moderate, 25-30, n (%) | 93 (27) | 47 (26) | 46 (27) |  |
| Moderately high, 31-35, n (%) | 44 (12) | 19 (11) | 25 (14) |  |
| High, >35, n (%) | 38 (11) | 18 (10) | 20 (12) |  |
| Depression Anxiety Stress Scale 21 |  |  |  |  |
| Depression, au, median (range) | 2 (0 to 36) | 2 (0 to 30) | 2 (0 to 36) | 0.96  0.80 |
| Normal, 0-9, n (%) | 283 (81) | 147 (82) | 136 (80) |  |
| Mild/moderate, 10-20, n (%) | 45 (13) | 21 (12) | 24 (14) |  |
| Severe/extremely severe, 21+, n (%) | 22 (6) | 11 (6) | 11 (6) |  |
| Anxiety, au, median (range) | 4 (0 to 34) | 4 (0 to 34) | 4 (0 to 30) | 0.49  0.87 |
| Normal, 0-7, n (%) | 240 (69) | 121 (68) | 119 (70) |  |
| Mild/moderate, 8-14, n (%) | 68 (19) | 35 (19) | 33 (19) |  |
| Severe/extremely severe, 15+, n (%) | 42 (12) | 23 (13) | 19 (11) |  |
| Stress, au, median (range) | 8 (0 to 36) | 8 (0 to 34) | 8 (0 to 36) | 0.82  0.88 |
| Normal, 0-14, n (%) | 257 (73) | 130 (73) | 127 (74) |  |
| Mild/moderate, 15-25, n (%) | 66 (19) | 35 (19) | 31 (18) |  |
| Severe/extremely severe, 26+, n (%) | 27 (8) | 14 (8) | 13 (8) |  |
| *Data presented as mean (standard deviation) unless otherwise indicated; Comparisons between groups conducted using chi-square (categorical variables) or linear regression (continuous variables). *p<0.05* | | | | |

**Table S5.** Demographic characteristics of included pregnant people (n=348).

|  | | | | |
| --- | --- | --- | --- | --- |
|  | All  (n=351) | Intervention  (n=179) | Usual Care  (n=172) | P Value |
| Gestational diabetes, n (%) | 26 (7) | 13 (7) | 13 (8) |  |
| Region, n (%) |  |  |  | 0.66 |
| Rural | 86 (25) | 42 (24) | 44 (27) |  |
| Urban | 252 (75) | 132 (76) | 120 (73) |  |
| Area Deprivation Index, median (range) |  |  |  |  |
| National rank, au | 69 (17 to 100) | 69 (24 to 100) | 68 (17 to 100) | 0.69 |
| State rank, au | 5 (1 to 10) | 5 (1 to 10) | 5 (1 to 10) | 0.67 |
| Sleep, min | 396 (78) | 393 (79) | 399 (77) | 0.55 |
| Time of enrolment, n (%) |  |  |  | 0.57 |
| Before March 2020 | 21 (6) | 12 (7) | 9 (5) |  |
| March 2020 – March 2021 | 45 (13) | 20 (11) | 25 (15) |  |
| April 2021 and after | 285 (81) | 147 (82) | 138 (80) |  |
| LDH region, n (%) |  |  |  | 0.84 |
| Region 1 | 43 (12) | 22 (12) | 21 (12) |  |
| Region 2 | 98 (28) | 50 (28) | 48 (28) |  |
| Region 3 | 43 (12) | 22 (12) | 21 (12) |  |
| Region 4 | 59 (17) | 30 (17) | 29 (17) |  |
| Region 5 | 23 (7) | 11 (6) | 12 (7) |  |
| Region 6 | 31 (9) | 16 (9) | 15 (9) |  |
| Region 7 | 7 (2) | 3 (2) | 4 (2) |  |
| Region 8 | 4 (1) | 4 (2) | 0 (0) |  |
| Region 9 | 43 (12) | 21 (12) | 22 (13) |  |
| *Comparisons between groups conducted using chi-square (categorical variables) or linear regression (continuous variables). LDH = Louisiana Department of Health.* | | | | |

**Table S6.** Household Chaos changes amongst body mass index categories and treatment groups (n=258).

|  | | | | | | |
| --- | --- | --- | --- | --- | --- | --- |
|  | n | Early Pregnancy | Late Pregnancy | Change | P Value |  |
| Body Mass Index Category |  |  |  |  |  |  |
| Normal, 18.5-24.9 kg/m^2^ | 89 | 25.76 (7.02) | 26.10 (7.68) | 0.40 (6.94) | 0.14 |  |
| Overweight, 25.0-29.9 kg/m^2^ | 76 | 25.64 (7.49) | 25.64 (7.44) | 0.00 (6.48) | 0.99 |  |
| Obesity, 30.0-40.0 kg/m^2^ | 93 | 26.45 (7.71) | 27.43 (9.94) | 0.97 (10.5) | 0.58 |  |
| Treatment Groups |  |  |  |  |  |  |
| Intervention | 135 | 25.54 (7.41) | 26.29 (9.01) | 0.74 (9.32) | 0.35 |  |
| Usual Care | 123 | 26.44 (7.37) | 26.65 (7.94) | 0.21 (6.98) | 0.73 |  |
| *Data presented as mean (standard deviation). Comparisons made using paired t-test.* | | | | | | |
